# T. J. Word, M. D., Rome, Ga.

**Published:** 1890-07

**Authors:** 


### T. J. WORD, M. D.

Dr. T. J. Word, of Rome, Ga., died there on the night of May 30th, after a long and severe illness. Dr. Word was the brother-in-law of Dr. H. V. Miller and an uncle of Dr. M. B. Hutchins. He had practiced his profession in Rome, Columbus and Atlanta, Ga., being associated here with Dr. Miller. He was a Christian gentleman, full of kind and generous impulses, skilled in his profession, and possessing many warm friends. He " died in the faith " and has passed into a well-deserved rest from cares of earth and the troublous ravages of disease.

Below we give brief extracts from Sanford, Fla., Rome, Ga., and New Orleans papers, in which are also described the death of Dr. Word's father at Iuka, Miss., and his uncle in Palestine, Texas. Father and son died the same day, the former at the age of ninety-four, and the latter almost at the allotted age of man.

From *Sanford Weekly Journal:*

Within a few days of each other there died recently three members of an old family, last representatives of a noble race. We write of Col. James Word, Iuka Springs, Miss., aged 94 years; Col. T. J. Word, of Palestine, Texas, aged 85 years, and

Dr. T. J. Word, of Rome, Ga., aged 64 years. Dr. Word was a surgeon in the Confederate army. His first wife, the mother of Mrs. Caldwell, was Miss Georgia Mitchell, daughter of Col. Mitchell, of Rome; his second wife, who survives him, was Mrs. G. W. R. Bayley, of New Orleans.

Dr. Word had been for some time a great sufferer, and with him dying was but entering into rest.

From Rome paper:

DEATH OF DR. T. J. WORD.

Friday night, 12:30 o'clock, Dr. T. J. Word, one of the oldest and most highly esteemed citizens of Rome, died at his residence on Third avenue.

Dr. Word was born in Gorgia and had been a resident of Rome for forty years. His death was not unexpected, and he died watched over by his loving relatives and friends.

The beautiful and simple faith of this venerable Christian, in his Heavenly Father, will ever be a source of comfort to those loved ones who feel so deeply the loss of him who was a devoted father and a friend to the friendless, and a Christian gentleman. The many noble deeds of Dr. Word had gathered around him friends whose grief at his death evidences the love and respect that his high character and lovable disposition commanded.

Dr. Word was 64 years of age and has three children living, Mrs. Frank Caldwell, of Sanford, Florida, and two sons who are citizens of another State.

Col. James Word, of Iuka Springs, Miss., father of Dr. Word, died Friday evening at forty minutes of three, just a few hours before the death of his son. Col. James Word was 94 years of age. Col. Thomas J. Word, an uncle of Dr. T. J. Word, died in Palestine, Tex., on the 26th. It is a remarkable coincidence that the death of these three prominent gentlemen should have occurred so closely together.

The funeral services of Dr. Word were conducted by Dr. Quillian at the First Methodist church yesterday afternoon.

